# Self-processing in coma, unresponsive wakefulness syndrome and minimally conscious state

**DOI:** 10.3389/fnhum.2023.1145253

**Published:** 2023-04-12

**Authors:** Fabrice Ferré, Lizette Heine, Edouard Naboulsi, Florent Gobert, Maude Beaudoin-Gobert, Frédéric Dailler, William Buffières, Alexandra Corneyllie, Benjamine Sarton, Béatrice Riu, Jacques Luauté, Stein Silva, Fabien Perrin

**Affiliations:** ^1^CAP Team (Cognition Auditive et Psychoacoustique), Lyon Neuroscience Research Centre (Université Claude Bernard Lyon 1, INSERM U1028, CNRS UMR5292), Bron Cedex, France; ^2^Intensive Care Unit, Purpan University Teaching Hospital, Place du Dr Joseph Baylac, Toulouse CEDEX 9, France; ^3^Toulouse NeuroImaging Centre (ToNIC), UPS—INSERM UMR, Place du Dr Joseph Baylac, Purpan University Teaching Hospital, Toulouse CEDEX 3, France; ^4^Neuro-Intensive Care Unit, Hospices Civils de Lyon, Neurological Hospital Pierre-Wertheimer, Bron, France; ^5^Trajectoires Team, Lyon Neuroscience Research Centre (Université Claude Bernard Lyon 1, INSERM U1028, CNRS UMR5292), Bron, France; ^6^Physical Medicine and Rehabilitation Department, Henry-Gabrielle Hospital, Hospices Civils de Lyon, Saint Genis Laval, France

**Keywords:** disorders of consciousness, coma, self-processing, event-related potentials, P300

## Abstract

**Introduction:**

Behavioral and cerebral dissociation has been now clearly established in some patients with acquired disorders of consciousness (DoC). Altogether, these studies mainly focused on the preservation of high-level cognitive markers in prolonged DoC, but did not specifically investigate lower but key-cognitive functions to consciousness emergence, such as the ability to take a first-person perspective, notably at the acute stage of coma. We made the hypothesis that the preservation of self-recognition (i) is independent of the behavioral impairment of consciousness, and (ii) can reflect the ability to recover consciousness.

**Methods:**

Hence, using bedside Electroencephalography (EEG) recordings, we acquired, in a large cohort of 129 severely brain damaged patients, the brain response to the passive listening of the subject’s own name (SON) and unfamiliar other first names (OFN). One hundred and twelve of them (mean age ± SD = 46 ± 18.3 years, sex ratio M/F: 71/41) could be analyzed for the detection of an individual and significant discriminative P3 event-related brain response to the SON as compared to OFN (‘SON effect’, primary endpoint assessed by temporal clustering permutation tests).

**Results:**

Patients were either coma (*n* = 38), unresponsive wakefulness syndrome (UWS, *n* = 30) or minimally conscious state (MCS, *n* = 44), according to the revised version of the Coma Recovery Scale (CRS-R). Overall, 33 DoC patients (29%) evoked a ‘SON effect’. This electrophysiological index was similar between coma (29%), MCS (23%) and UWS (34%) patients (*p* = 0.61). MCS patients at the time of enrolment were more likely to emerged from MCS (EMCS) at 6 months than coma and UWS patients (*p* = 0.013 for comparison between groups). Among the 72 survivors’ patients with event-related responses recorded within 3 months after brain injury, 75% of the 16 patients with a SON effect were EMCS at 6 months, while 59% of the 56 patients without a SON effect evolved to this favorable behavioral outcome.

**Discussion:**

About 30% of severely brain-damaged patients suffering from DoC are capable to process salient self-referential auditory stimuli, even in case of absence of behavioral detection of self-conscious processing. We suggest that self-recognition covert brain ability could be an index of consciousness recovery, and thus could help to predict good outcome.

## Introduction

The assessment of coma and other disorders of consciousness (DoC), following severe brain injury, is extremely challenging. The central issue is both the evaluation of sensory-motor and cognitive functions but also awareness of self and the environment. The latter are currently inferred on the basis of the patient’s behavioral reactivity and is the backbone of the diagnostic classification (Giacino et al., 2004). Coma is a state of profound unawareness from which the patient cannot be aroused and is defined by an absence of eye opening and adapted motor response even after nociceptive stimuli (Plum and Posner, 1983). Following a coma, a patient regaining an eye-opening/closing cycle and reflexive motor activity, devoid of any voluntary interaction with the environment, is diagnosed in a unresponsive wakefulness syndrome (UWS, formerly known as vegetative state; Laureys et al., 2010). The diagnosis of minimally conscious state (MCS) is proposed for patients who are able to produce reproducible but inconsistent non-reflexive behaviors (e.g., visual pursuit, reproducible movement to command; Giacino et al., 2004; Giacino, 2005; Laureys et al., 2009; Rohaut et al., 2013, 2019). The emergence from MCS (EMCS) is established if the patient is capable of accurate communication or functional use of objects (Giacino et al., 2002).

The diagnosis of UWS, MCS and EMCS requires the practical use of the revised version of the Coma Recovery Scale (CRS-R), which is now considered as the gold standard (Giacino et al., 2004, 2018; Schnakers et al., 2008a; Kondziella et al., 2020). However, it is now well known that the behavioral description of these patients does not systematically reflect their residual brain or cognitive functions (Fernandez-Espejo and Owen, 2013; Schnakers et al., 2022). Dissociations between behavior and brain activity have been observed repeatedly, both with fMRI and Electroencephalography (EEG) methodologies, in various very simple or complex protocols. The most popular study is undoubtedly that of Owen and colleagues in which they showed that a UWS patient showed brain activity comparable to that of control subjects during mental imagery and command-following tasks (Owen et al., 2006). This observation is exceptional probably because the cognitive functions of interest are complex (Monti et al., 2010). Nevertheless, by measuring lower-level cognitive processes, it has been also shown that a larger number of DoC patients, probably around 15% of them (Kondziella et al., 2016; Schnakers et al., 2020), may exhibit such dissociations. For example, studies using passive language and/or music stimuli have shown that some patients with DoC demonstrate association cortex responses despite absent behavioral evidence of language comprehension [Coleman et al., 2009; Okumura et al., 2014; Edlow et al., 2017; for a systematic review of residual implicit language abilities during passive language listening tasks in patients with DoC, see Aubinet et al. (2022)]. Taken together, these studies are extremely important since they suggest that brain activities associated with cognitive functions, and sometimes probably with consciousness, can be observed in patients for whom the behavior rather suggests its failure.

In the context of non-communicative patients, it is useful to know whether they respond (cerebrally and/or behaviorally) to their own name. Indeed, the presence of such a response means that he/she can detect or discriminate a self-referential stimulus, i.e., an item of the environment that refers to her/him (Fingelkurts and Fingelkurts, 2023). Its presence suggests not only the preservation of one aspect of the self but also a possible perspective taking, i.e., meta-representations of mental and bodily states as one’s own mental and bodily states (Vogeley and Fink, 2003). Dissociations between brain and behavior responses to the own name have been reported in patients with DoC. Note that these observations are possible because the EEG cerebral response to one’s own name is strong enough to be studied at the individual level. For example, Perrin et al. (2006) had shown that the cerebral response to one’s own name (versus unfamiliar other first names) was observed in 3/5 of UWS patients (and 6/6 MCS) while they had no behavioral response to this stimulation (Perrin et al., 2006). Its presence in UWS and MCS patients has been confirmed in other studies, but always in small cohorts of patients (Perrin et al., 2006; Castro et al., 2015; Heine et al., 2021). Thus, no cohort study has investigated the percentage of UWS or MCS patients who show this response and whether it is also observable in comatose patients.

The electrophysiological response to one’s own first name is observed in different states of unconsciousness, in sleep (Perrin et al., 1999) and using subliminal presentation (Doradzinska et al., 2020). Thus, it may not reflect self-awareness but rather a self-processing, i.e., the ability to probe an autobiographical memory. If it is true, this response should be observable in DoC, including coma, i.e., regardless of the patient’s behavioral ability and with a similar probability of occurrence regardless of diagnosis (coma, UWS and MCS).

If the brain response to the subject’s own name is not a sign of awareness, it could rather reflect the persistence of a mechanism that is essential for the recovery of consciousness. Indeed, it is often admitted empirically that self-processing would be a prerequisite for consciousness: “Experience is impossible without an experiencer” (Damasio, 2003; Lane, 2020). Interestingly, Damasio investigated minimal forms of self that he coined ‘mental or core self’ stipulating that they are required in the making of consciousness (Damasio, 1999). If self is necessary for consciousness, then the presence of a cerebral response to the patient’s own name should be associated with a very high rate of favorable evolution (whereas its absence could indicate nothing since the response could reappear later). In line with this hypothesis, Castro et al. (2015) observed a link between patients with a brain response to their own name and a favorable patient outcome, but the authors could not conclude because of the small cohort of patients (Castro et al., 2015).

Through the study of electrophysiological marker of auditory discrimination of the subject’s own name in a large cohort of coma, MCS and UWS patients with a documented outcome, we made the assumption that a P3 (aka P300) response could be identified independently of their behavioral status, and that its presence would be associated with a favorable outcome.

## Materials and methods

### Population

Electrophysiological data (i.e., ERPs induced by the subject’s own name (SON) auditory task) from 22 healthy subjects (mean age 34.5 years (± 14.7), sex ratio (M/F): 14/8, right-handed, postgraduate) were recorded from February 2017 to June 2018. Coma, MCS and UWS patients hospitalized in the Critical Care Unit of the University Hospital of Purpan (Toulouse, France) between December 2017 and October 2019 or hospitalized in the Critical Care Unit or in the Post-Critical Care Neurological Rehabilitation Unit of the Pierre Wertheimer Hospital (Hospices Civils de Lyon, Bron, France) during the 2011–2022 period were included in the present study. During their stay, several evaluations and exams were performed when indicated including neurological clinical assessment, brain CT scan and structural brain MRI, clinical EEG, and ERPs induced by the subject’s own name (SON) auditory task. The study was approved by the ethics committees “CPP Sud-Est II (2012–036-2)” and “CPP Nord-Ouest II (69LHCL19_0672).” Written consent was obtained from healthy participants and all patients’ close relatives. All experiments were conducted in accordance with the guidelines of the Declaration of Helsinki.

### Clinical assessment of behavioural diagnosis and outcome

#### Diagnosis

The state of coma of acute severely brain-damaged patients was determined using both of the following criteria: GCS ≤ 8 and absence of eye opening and adapted motor response even after nociceptive stimuli at the time of enrolment (Rohaut et al., 2019). The UWS or MCS behavioral states of consciousness were determined by neurologists or intensivists (FF, EN, FG, FD, WB, BS, BR, JL, and SS) who were trained users of the French version of the CRS-R (Giacino et al., 2004; Schnakers et al., 2008a). We used the CRS-R score measured immediately before the SON task ERP recording. In case of discrepancy with previous CRS-R scores, a consensus-based diagnosis was applied. Interruption of any sedative agent for at least 48 h (for propofol, ketamine, clonidine, morphine, dexmedetomidine) or 72 h (for benzodiazepines) was a prerequisite for the ERPs recording.

#### Outcome

The primary outcome was patient status assessed 6 months after the brain injury and was collected by trained users of the CRS-R during an in-person neurological clinical assessment realized by neurologists or specialists of neurorehabilitation (for patients still in rehabilitation centers), or by one of the study investigators through a dedicated in-person visit (when appropriate) or, alternatively, through a structured phone interview with patient’s relatives who were questioned about items derived from the CRS-R (motor, visual, auditory, oromotor and communication functions scale) and items of the daily life. An item was considered as present only when the corresponding behavior was univocal. Two measures of recovery have been evaluated: conscious state and behavioral improvement. Patients were considered to have recovered consciousness if they were categorized EMCS (i.e., univocal functional use of object or accurate communication; Giacino et al., 2004) at 6 months. Behavioral improvement was stipulated for patients who were in a coma at the inclusion and were MCS or EMCS at 6 months, for patients who were in a UWS at the inclusion and were MCS or EMCS at 6 months, and for patients who were in a MCS at the inclusion and were EMCS at 6 months. The Glasgow outcome scale (GOS) defining 5 categories (from 1 = death to 5 = good recovery) of possible outcomes after a brain injury was also collected at the same time (Jennett and Bond, 1975).

### Subject’s own name paradigm: Stimuli and procedure of ERPs recordings

From 2011 to 2022, three different versions (v1, v2, and v3; please see supplementary Text for details) of the SON paradigm were developed and tested, and part of these data were previously published (Castro et al., 2015; Heine et al., 2021). The common main aim of these protocols was to investigate the cerebral discriminative response to the SON against 7 (v1) or 6 (v2 and v3) irrelevant stimuli (other unfamiliar first names; OFN). The SON (or nickname if relevant) was selected for each subject. Irrelevant OFN were selected by asking participants or representatives to indicate on a predefined list if any were familiar or not. All OFN were disyllabic (1.05 s, SD = 0.05 s). All names were pronounced by a female voice (v1) or by voice(s) created using text to speech software with a neutral intonation (Natural Reader, NaturalSoft Ltd.). All stimuli were equalized to the same A-weighted sound level, and presented binaurally during the experiment at a sound pressure level of approximatively 65 dBA SPL. In patients, if environmental noise was high, the presentation level was slightly increased to a level that was clearly audible but not painful.

Ten sequences of 64 equiprobable first names (v1), or 24 sequences (v2) or 12 sequences (v3) of 42 first names, were created and presented in a pseudo-random order (with no repetition of a same name and with a homogeneous temporal distribution of the first names). The mean stimulus onset asynchrony was 1,414 ± 137 ms (v1) and was between 1,400 and 1,500 ms, with random steps of 100 ms (v2 and v3). The three versions also varied by the presence (or not) of excerpt of music that preceded each sequence of first name, and we decided to average the first names after music and its control condition (neutral sound) to enhance the signal to noise ratio (and because very minor differences exist between the averages after all contexts, music + control, and the averages after music).

Finally, all subjects were instructed as follows: “You will hear a series of names […]. You will hear them passively but you must pay attention. The experiment lasts about [x] minutes”; [x] depending on the version of the protocol.

### EEG recording and preprocessing

EEG signals were acquired in v1 from 13 Ag/AgCl electrodes referenced to the nose, as well as a bipolar EOG (below and above the right eye) and amplified using SystemPlus EEG amplifier (Micromed^®^) and in v2 and v3 from 128 electrodes referenced to the vertex and amplified using geodesic sensor net (EGI^®^, Philips) system.

All raw data were resampled at 250 Hz and visually inspected to identify bad channels. Any channels with huge continuous outliers were indicated as bad, interpolated (using spherical spline method) but taken out of the analysis. Data were bandpass filtered between 0.1 and 40 Hz using a FIR zero-double filter and a notch at 50 Hz. For patients, a second analysis was done with data filtered between 1 and 40 Hz [as previously motivated in (Sergent et al., 2017; Heine et al., 2021)] and an effect was suggested if one of the two analyses showed an effect. All electrodes signal were calculated from an average reference. Cz was interpolated for EGI recordings. For any subject where data were affected by eye-blinks, an ICA (*fastICA*) was performed to remove the blink components from the signal. Trials were then segmented (epochs) from − 200 ms to + 1,000 ms relative to the onset of the stimulus and a baseline correction (− 200 to 0 ms) was applied. To further clean the data, an automatic rejection function was used where bad trials are either interpolated or rejected based on trial-wise assessment of individual sensor thresholds (Jas et al., 2018). All these processing stages were performed using MNE-Python version 19.2.

### Event-related individual analyses

Averaged responses to SON and OFN preceding it (for comparisons with similar signal-to-noise ratio) were computed for each individual and for each of the 13 electrodes common to the Micromed^®^ and EGI^®^ acquisition systems. Statistical differences between SON and OFN were tested at the individual level (for healthy participants and DoC patients), using temporal clustering permutation tests, with one sided *t*-tests and 10,000 permutations (Maris and Oostenveld, 2007). Cluster level alpha was set to 0.01 with a cluster forming threshold of 0.05. To reduce the risk of false discovery rate by making multiple comparisons on 13 electrodes, a ‘SON effect’ (defined as the statistical difference between ERP elicited in SON and OFN conditions) was deemed present if a temporal cluster was identified on at least 2 electrodes (whether they were contiguous or not) from 200 ms after stimulus onset to the end of epoch. This criterion was determined on the basis of the large time window effect observed between SON and OFN in previous studies (Perrin et al., 2006). The minimal duration for a significant temporal cluster was measured at 48 msec.

### Comparison between ERPs effect and outcome

The normality of quantitative data was verified using the Shapiro–Wilk test. Quantitative data were expressed as median (25th–75th percentile) or mean (± standard deviation) as appropriate. Qualitative variables were expressed as number (%). Categorical variables were compared using Chi2 or McNemar tests. Frequentist approach was used to compute sensitivity (Se), specificity (Sp), positive predictive value (PPV), negative predictive value (NPV), positive (LR+), negative (LR-) likelihood ratio, and area under the receiver operating characteristic curve (AUC). Statistical analysis was performed using MedCalc software (version 12.6.1, MedCalc Software bvba, Ostend, Belgium; 2013). A value of *p* < 0.05 was considered statistically significant.

## Results

During the 2011–2022 period, 129 non-communicating patients were recorded with ERPs acquisition during the SON paradigm. Seventeen patients were excluded because of insufficient electrophysiological data quality. The final cohort consisted of 112 patients, age 46.0 (±18.3) years, of whom 71 (63%) were males (Table 1).

**Table 1:** Clinical and demographics characteristics of patients with disorders of consciousness (overall population).

	Patients (*n* = 112)
Age (years)	46 ± 18.3
Age ≥ 45 years	62 (55.4%)
Sex ratio (M/F)	71/41
Diagnosis	
Coma	38 (33.9%)
MCS	30 (26.8%)
UWS	44 (39.3%)
Etiology	
TBI	55 (49%)
Others	57 (51%)
Anoxia	32
ICH	14
Metabolic	5
Ischemic	4
Tumoral	1
Encephalitis	1
Delay since brain injury (days)	23 [14–49]
Acute (≤ 1 month)	71 (63.4%)
Acute and subacute (≤ 3 months)	99 (88.4%)
Patients with a SON effect	33 (29.5%)
Outcome at 6 months (*n* = 109)	
GOS (/5)	3 [2–3]
EMCS, MCS, UWS, dead	48, 20, 17, 24
Recovery of consciousness (i.e., EMCS)	48 (44%)

Data are expressed as *n* (%), mean (± SD) or median [25th–75th percentile] as appropriate.

M = male; F = female; MCS = minimally conscious state; UWS = unresponsive wakefulness syndrome; TBI = traumatic brain injury; ICH = intracerebral hemorrhage; ERP = event-related potential; SON = subject’s own name; GOS = Glasgow outcome scale; EMCS = emergence of MCS

Among these 112 patients, 38 (34%), 30 (27%) and 44 (39%) were in a state of coma, MCS or UWS, respectively (Table 1). The most common etiology was traumatic brain injury (TBI; 49%), then anoxia (29%). The delay between the brain lesion and the evaluation was ≤ 3 months for 99 patients (88%; Table 1).

### Event-related potentials to SON

Seventeen of the 22 healthy subjects (77%) had a significant effect between SON and OFN. Illustration of the statistically significant P3 event-related potential in response to SON versus OFN at the group level is available in Supplementary Figure.

**Figure 1:**
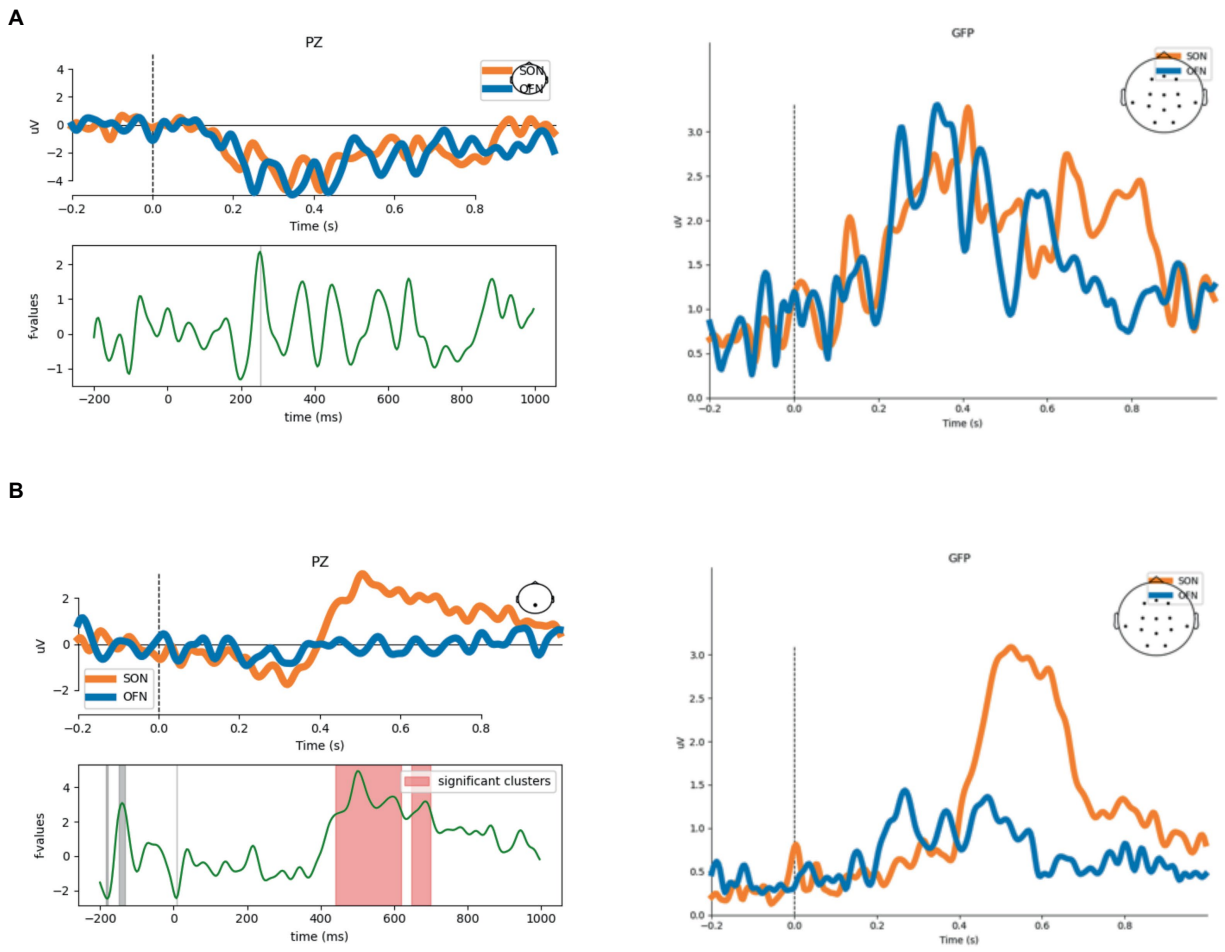
Illustrative cases. Event-related potentials (ERP) at Pz and global field power (GFP) from two patients are represented: one COMA patient without SON effect **(A)**, and one COMA patient with SON effect **(B)**. Temporal clustering permutation tests, with one sided *t*-tests and 10,000 permutations. Significance threshold: alpha cluster was set to 0.01; value of *p* ≤ 0.05 for SON (orange curve) and OFN (blue curve) comparison at each sample. Abbreviations: SON = subject’s own name; OFN = other first names.

Thirty-three patients (29%) had a statistically significant different brain response between SON and OFN conditions. Interestingly, no difference in the incidence of this SON effect was found between coma (11/38, 29%), MCS (7/30, 23%) and UWS patients (15/44, 34%; *p* = 0.61 for comparison between groups). Furthermore, the effect was more frequently observed in non-traumatic brain patients (23/57, 40%) than in traumatic brain patients (10/55, 18%; *p* = 0.01 for comparison between groups). Cases of patients with and without a SON effect are illustrated in Figure 1.

### Outcome of DoC patients

#### Overall population of DoC patients

Among the 109 patients with a documented outcome, 85 patients (78%) survived 6 months after the brain damage. Concerning their behavioral evolution, 48 patients (44%) were EMCS, 20 patients (18%) were MCS, and 17 patients (16%) were UWS at 6 months (Table 1). MCS patients at the time of enrolment were more likely to recover consciousness at 6 months (20/30, 67%) than coma (13/36, 36%) and UWS patients (15/43, 35%; *p* = 0.013 for comparison between groups). The median (25th–75th percentile) GOS was 3 (2–3). The global outcome of traumatic brain patients was better than non-traumatic brain one [respectively 33 patients (62%) vs. 15 patients (26%) were EMCS at 6 months, *p* = 0.0002].

#### Survivors

**Figure 2:**
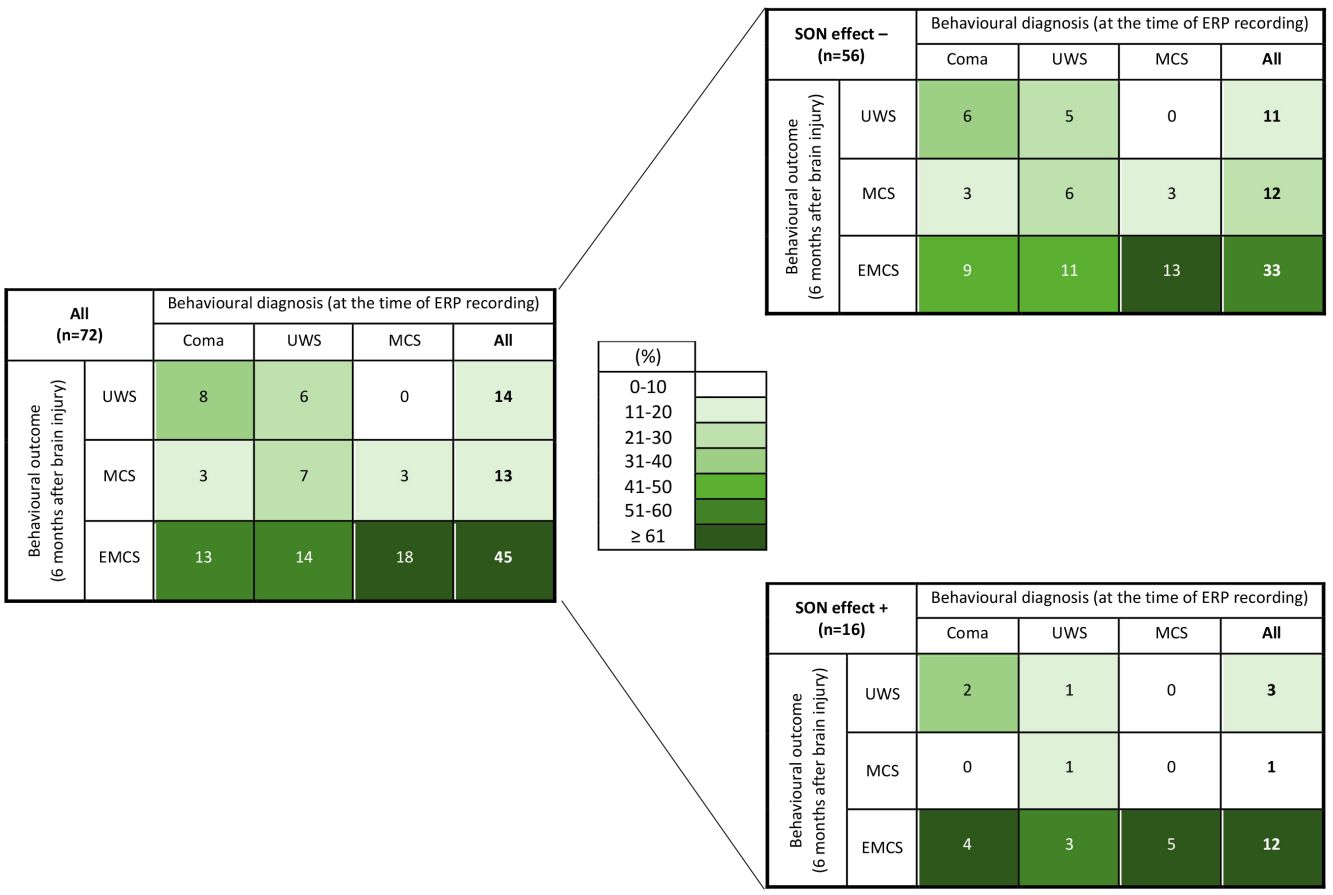
‘Heat maps’ illustrating 6 months behavioral outcome of survivors’ patients with disorders of consciousness according to their behavioral state of consciousness at the time of ERP recording and the absence/presence of a SON effect at the acute stage of brain injury. Background color coding indicates density of patients (%) within a diagnostic category, suggesting clusters of observations. UWS = unresponsive wakefulness syndrome; MCS = minimally conscious state; EMCS = emergence of minimally conscious state; SON = subject’s own name; SON effect −/+ = absence/presence of a SON effect.

**Figure 3:**
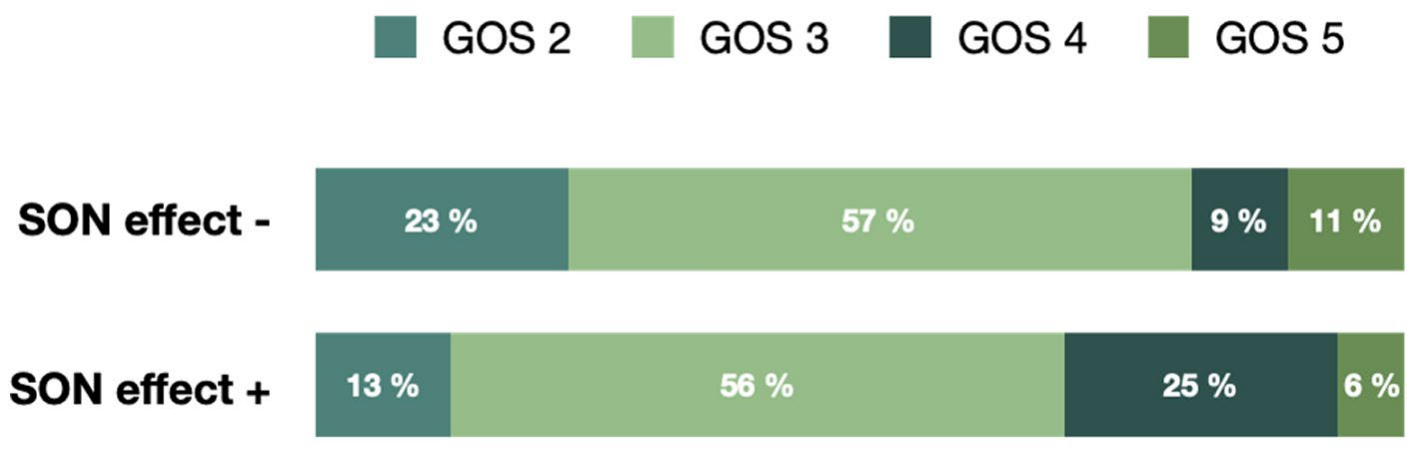
Glasgow Outcome Scale of survivors’ patients with disorders of consciousness 6 months after their brain injury according to the absence/presence of a SON effect at the time of ERP recording. GOS = Glasgow outcome scale; SON = subject’s own name; GOS 2/3/4/5: persistent vegetative state/severe disability/moderate disability/good recovery.

The analyses of the predictive power of a SON effect were conducted on survivors to mitigate the impact of withdrawing of life-sustaining therapies in potentially conscious but extremely impaired patients (Perez et al., 2020). Hence, the behavioral outcome at 6 months regarding the presence/absence of a SON effect has been studied in the 72 survivors’ patients for whom the delay between brain injury and EEG recording was ≤ 3 months (‘acute and subacute patients’; Figures 2, 3). Among them, 45 (63%) were EMCS at 6 months. Concerning the 16 (22%) patients with a SON effect, 75% of them were EMCS at 6 months, while 59% of the 56 (78%) patients without a SON effect were EMCS at 6 months. In other words, the false positive (percentage of unconscious patients at 6 months among patients with a SON effect) and false negative (percentage of conscious patients at 6 months among patients without a SON effect) rates were 25 and 59%, respectively. The prognostic value (Se, Sp, PPV, NPV, LR +, LR – and AUC) of the SON effect in DoC patients are reported in Supplementary Table 1 (for recovery of consciousness) and Supplementary Table 2 (for behavioral improvement).

#### Focus on coma patients

The characteristics of the 38 coma patients are detailed in Table 2. Individual analyses showed 11/38 patients (29%) with a SON effect. Among the 36 coma patients with a documented outcome, 24 of them (67%) were alive at 6 months among whom 13 (54%) were EMCS (Table 2; Figure 2). Concerning the 6 (25%) survivors’ patients with a SON effect, 67% of them were EMCS at 6 months, while 50% of the 18 patients (75%) without a SON effect were EMCS at 6 months. In other words, the false positive and false negative rates were 33 and 50%, respectively. The prognostic value (Se, Sp, PPV, NPV, LR +, LR– and AUC) of the SON effect in coma patients are reported in Supplementary Table 3 (for recovery of consciousness) and Supplementary Table 4 (for behavioral improvement).

**Table 2:** Clinical and demographics characteristics of coma patients.

	Coma patients
(*n* = 38)	
Age (years)	49.6 ± 19.9
Age ≥ 45 years	25 (65.8%)
Sex ratio (M/F)	21/17
Etiology	
TBI	15 (39.5%)
Others	23 (60.5%)
Delay since brain injury (days)	14.5 [10–24]
Acute (≤ 1 month)	33 (86.8%)
Acute or sub-acute (≤ 3 months)	38 (100%)
Patients with a SON effect	11 (28.9%)
Outcome at 6 months (*n* = 36)	
GOS (/5)	2 [1–3]
EMCS, MCS, UWS, dead	13, 3, 8, 12
Recovery of consciousness (i.e., EMCS)	13 (36.1%)

Data are expressed as *n* (%), mean (± SD) or median (25th–75th percentile) as appropriate.

M = male; F = female; TBI = traumatic brain injury; SON = subject’s own name; GOS = Glasgow outcome scale; EMCS = emergence of MCS; MCS = minimally conscious state; UWS = unresponsive wakefulness syndrome.

## Discussion

The neurological outcome following severe brain injury is a daily interrogation for the caregivers and family members of (acute) non-communicating patients. Clinicians specializing in the care of severely brain-damaged patients are well acquainted with the clinical features of DoC. Notably, coma and UWS patients are characterized by the complete absence of behavioral signs of self and environmental awareness, the likelihood of withholding life-sustaining therapies or denying rehabilitative services increasing substantially with the persistence of this behavioral status. In this context, we reported new evidence about covert abilities to discriminate self-relevant words in this specific population of patients. Interestingly, we demonstrated, in a large series of comatose and other DoC patients, that the presence of a bedside differential brain response to the SON could help to predict behaviorally overt consciousness recovery, questioning the role of this cerebral index as potentially being a key-cognitive function to consciousness emergence (Lane, 2020).

The use of personally relevant stimuli has been promoted in recent years for investigating severely brain damaged DoC patients with the aim of identifying their ability to categorize self-related stimuli (Perrin et al., 2006; Castro et al., 2015; Perrin et al., 2015). In this context, it has been demonstrated that hearing one’s own first name, presented within other unfamiliar first names, evoked a P3 potential in some patients (Perrin et al., 2006). In our study, we demonstrated that coma, MCS and UWS patients were able to discriminate their own name (compared to unfamiliar first names) as a significant P3 was individually observed in about 30% of them. Interestingly, this SON effect has no added value in clarifying the diagnosis of an altered state of behavioral awareness. Indeed, whereas several published articles supposed that it should be mainly found in MCS patients in which definite behavioral evidence of self-awareness is demonstrated (Perrin et al., 2006; Schnakers et al., 2008b, 2015; Hauger et al., 2015; Sergent et al., 2017)—for a review, see (Wutzl et al., 2021)—a P3 response to SON was indifferently observed in all the phenotypes of patients. This result paves the way of a potentially existing dissociation between electrophysiological evidence of self and environmental processing and the complete absence of its behavioral signs, notably in the acute stage of coma and in UWS patients. In the early 2000s, functional neuroimaging studies suggested that cognitive processing capacity might be underestimated in MCS patients (Hirsch et al., 2001; Schiff et al., 2005). Based on our results, we assume that cortical brain activity that is dissociated from behavior is possible in patients with UWS (Edlow et al., 2017), but also in the acute stage of coma. The pattern of residual neural activity of a self-related stimulus perceptive discrimination we identified for the first time in such individuals suggest that EEG paradigms are required to complement behavioral assessment in patients without command following at the bedside (Kondziella et al., 2020). Definitely, behavior is an indirect and thus incomplete measure of brain functions leading to interpretative errors in these patients. Consequently, standardized clinical evaluation and neuroimaging-based measures (including bedside EEG-based techniques) should be integrated for multimodal evaluation of patients with DoC in accordance with the guidelines of the European and American Academies of Neurology on the diagnosis of coma and other DoC (Giacino et al., 2018; Kondziella et al., 2020).

Current conceptual models of consciousness, such as the global neuronal workspace theory (GNWT; Dehaene and Changeux, 2011) and the information integration theory (Tononi, 2004), propose that consciousness requires the integrated activity of association cortices. However, such activation is likely necessary but not sufficient for consciousness. Here, we do not assert that higher-order cortex motor dissociation is indicative of covert consciousness. In contrast, the preservation of self-recognition could reflect the ability to use first-person perspective and could be considered as a key-cognitive prerequisite to consciousness emergence. Indeed, one of our main results was the good positive predictive value attributed to the presence of a P3 component in response to SON: this self-recognition electrophysiological pattern could predict an improvement of consciousness until its behaviorally overt emergence. This specific brain reactivity to the own name alludes to Zeman’s fourth sense of self-consciousness referring as self-recognition, i.e., our ability to recognize our own bodies as our own, for example in mirror (Zeman, 2001, 2005). From a conceptual point of view, Northoff assumes self-referential processing, accounting for distinguishing stimuli related to one’s own self from those that are not relevant to one’s own concerns, to be at the core of the self (Northoff and Bermpohl, 2004; Northoff et al., 2006). Furthermore, Damasio investigated minimal forms of self that he coined ‘mental or core self’ stipulating that they are required in the making of consciousness (Damasio, 1999). In this setting, minimal self could be considered as the bifurcation point between conscious and unconscious states (Lane, 2020), and might constitute the basis for higher-level, cognitive forms of self, as well as the understanding of other minds (Limanowski and Blankenburg, 2013).

Finally, we assert that this covert brain ability to correctly categorize self-referential ecological stimulation from outside could be both an index of self-processing and a prerequisite for consciousness recovery. This brain ability observed in up to 30% of coma patients suggests that coma is not a passive state of sensory isolation, but rather a transient and active state that could benefit from a rich sensory stimulation regimen in which, from instance, music—and its autobiographical characteristics—could have a role to play through cortical arousal and/or awareness enhancement [in agreement with “the arousal and mood hypothesis” (Janata et al., 2007; Zatorre, 2013; Castro et al., 2015)].

Our results must be interpreted with caution and a number of limitations should be borne in mind. Firstly, the proof of a potential for consciousness recovery (or not) using electrophysiological biomarker of self-processing is forcefully being challenged at the individual level by the very weak negative predictive strength—meaning that its absence was not a reliable predictor of negative outcome–, the low sensitivity and also the wide PPV confidence interval we noticed. It is worth noting that the sensitivity for all cognitive evoked potentials is known to be low (i.e., with a high rate of false negative), even in healthy subjects (Perrin et al., 2006; Schnakers et al., 2008b; Fischer et al., 2010; Faugeras et al., 2011, 2012; Sergent et al., 2017). Nonetheless, a SON effect was detected in 17 of the 22 healthy subjects (77%) enrolled in our study. This relatively low rate of false negative could be due to the extreme salience of being presented one’s own name and might be associated to the enhancement of top-down and/or arousal mechanisms (Chennu and Bekinschtein, 2012). However, even if personal and emotional significance increases the probability to observe a brain response in DoC patients—P3 to SON is elicited more frequently as compared to P3 to rare tone (Cavinato et al., 2011)—the high rate of false negative (59%) in our cohort of patients underscores the need for caution in interpreting negative findings on EEG and encourages finding ways to improve the sensitivity of the SON paradigm (Castro et al., 2015). In this setting, we think that the very weak predictive strength of a negative effect could encourage to repeat the electrophysiological evaluation (longitudinal follow-up), the late recovery of a discriminative response to SON being theoretically possible. Whether the SON effect recovery is strongly associated with consciousness emergence would deserve to be studied. Moreover, complementary pattern of predictive power would be interesting. For instance, the ‘local effect’ (i.e., MMN/P3a obtained to local deviant sounds during the local–global auditory task) could be used as a surrogate marker of low-level perceptive function therefore reflecting the preservation of a local cortical network and playing as a necessary but insufficient condition to consciousness recovery (Baars, 1988; Dehaene and Naccache, 2001; Dehaene and Changeux, 2011). To go further, the use of a multifaceted ERP battery exploring more distinct cognitive processes to provide a more nuanced cognitive profile, from low-level perceptive (e.g., echoic memory) to higher-order cognitive abilities, would be promising (Sergent et al., 2017). Secondly, we were surprised to find a higher incidence of the P3 response to SON in non-traumatic (40%) than traumatic brain (18%) patients, while the latter had a more favorable neurological outcome. These findings could support the notion of brain cortical modularity in P3 generation to target stimuli. Theoretically, global forms of brain injury (e.g., cerebral hypoxia, diffuse axonal injury) could sever the connections between each module without destroying the module itself (Giacino, 2005). Under these circumstances, the functional integrity of a particular module (e.g., the module generating a P3 to self-relevant stimuli) may be spared. Thirdly, an accurate categorization of MCS patients into MCS + and MCS – subgroups and their respective SON effect would deserve to be investigated. Based on our results, we assume that no difference would be expected between these 2 categories of patients because we believe that the preservation of a (minimal) self-processing is independent of the behavioral impairment of consciousness. The minimal self almost certainly depends on brain processes and an ecologically embedded body, but one does not have to know or be aware of this to have an experience that still counts as a self-experience (Gallagher, 2000). Finally, modules that remain active but become isolated may produce higher-order cortical response that occur in the absence of conscious experience (please, see the intriguing possibility of ‘words without mind’ suggesting activity of isolated ‘islands of cortex’ described by Schiff et al. in a patient suffering from UWS (Schiff et al., 1999). Conversely, traumatic brain injury could produce a focal lesion of a specific cognitive module that may become underactive while connections between modules are spared. It would seem therefore interesting to confront our assumption with the topography of the traumatic brain lesions. Lastly, the calculation of the predictive strength of the SON effect on consciousness recovery was focused on long-term survivors in order to discard the impact of withdrawing of life-sustaining therapies in potentially conscious but extremely impaired patients. However, this methodological choice is not transposable to the management of patients and their family in real-time clinical practice.

To conclude, about 30% of severely brain-damaged patients suffering from DoC are capable to discriminate salient self-referential auditory stimuli, even in case of absence of behavioral detection of conscious processing. We suggest that this covert brain ability, detected for the first time in coma patients, could be both an index of self-processing and a prerequisite for consciousness recovery. Guide the research on the attentional modulation of the cortical discriminative response to the SON of non-communicating patients would contribute to enriching the discussion regarding neural correlates of access to pre-conscious and conscious content.

## Data availability statement

The raw data supporting the conclusions of this article will be made available by the authors, without undue reservation.

## Ethics statement

The studies involving human participants were reviewed and approved by CPP Sud-Est II (2012-036-2) and CPP Nord-Ouest II (69LHCL19_0672). The patients’ relatives/participants gave a written informed consent to participate in this study.

## Author contributions

FP contributed to conception and design of the study. FF, LH, AC, and FP contributed to analyses. FF, LH, SS, and FP contributed to interpretation of the results. FF wrote the first draft of the manuscript. All authors have contributed to data acquisition. All authors contributed to the article and approved the submitted version.

## Funding

This work was supported by the grant CogniComa (ANR-14-CE-15-0013) and the LabEx CeLyA (ANR-10-LABX-0060/ANR-16-IDEX-0005).

## Conflict of interest

The authors declare that the research was conducted in the absence of any commercial or financial relationships that could be construed as a potential conflict of interest.

## Publisher’s note

All claims expressed in this article are solely those of the authors and do not necessarily represent those of their affiliated organizations, or those of the publisher, the editors and the reviewers. Any product that may be evaluated in this article, or claim that may be made by its manufacturer, is not guaranteed or endorsed by the publisher.
