# Supplementary material for: Self-processing in coma, unresponsive wakefulness syndrome and minimally conscious state
Source: Front Hum Neurosci. 2023 Apr 12;17:1145253. doi: 10.3389/fnhum.2023.1145253 (PMC10132704; doi:10.3389/fnhum.2023.1145253)
Supplement: Supplementary file 1 [file Data_Sheet_1.docx]

***SUPPLEMENTARY MATERIAL***

**Self-processing in coma, unresponsive wakefulness syndrome and minimally conscious state**

Fabrice Ferré^1,2,3^, Lizette Heine^1^, Edouard Naboulsi^2^, Florent Gobert^1,4,5^, Maude Beaudoin^6^, Frédéric Dailler^4^, William Buffières^2,3^, Alexandra Corneyllie^1^, Benjamine Sarton^2,3^, Béatrice Riu^2^, Jacques Luauté^6^, Stein Silva^2,3^, Fabien Perrin^1^

^1^ CAP Team (Cognition Auditive et Psychoacoustique), Lyon Neuroscience Research Centre (Université Claude Bernard Lyon 1, INSERM U1028, CNRS UMR5292), 95 boulevard Pinel, 69675 Bron Cedex, France.

^2^ Intensive Care Unit, Purpan University Teaching Hospital, Place du Dr Joseph Baylac, 31059 Toulouse CEDEX 9, France.

^3^ Toulouse NeuroImaging Centre (ToNIC), UPS - INSERM UMR1214. Purpan University Teaching Hospital, Place du Dr Joseph Baylac, 31024 Toulouse CEDEX 3, France.

^4^ Neuro-Intensive care unit, Hospices Civils de Lyon, Neurological hospital Pierre-Wertheimer, 59 Boulevard Pinel, 69677 Bron, France.

^5^ Trajectoires Team, Lyon Neuroscience Research Centre (Université Claude Bernard Lyon 1, INSERM U1028, CNRS UMR5292), 16 avenue Doyen Lépine, 69676 Bron, France.

^6^ Physical Medicine and Rehabilitation Department, Henry-Gabrielle Hospital, Hospices Civils de Lyon, 69230 Saint Genis Laval, France.

**Corresponding author:**

Fabrice Ferré

Intensive Care Unit

Purpan University Teaching Hospital

Place du Dr Joseph Baylac, 31059 Toulouse CEDEX 9, France

E-mail: fabriceferre31@gmail.com

**Supplementary Text, Figure and Tables**

**Supplementary Text**

- - From 2011 and 2022, three different versions of the Subject’s Own Name (SON) paradigm (v1, v2 and v3) were developed and tested on patients with disorders of consciousness. In all three versions of the paradigm, SON and irrelevant stimuli (i.e., other unfamiliar first names; OFN) were equiprobably presented, thus guaranteeing that the evocation of a P3 ERP response was related to self-processing (Perrin et al., 2006; Castro et al., 2015; Heine et al., 2021).
  - The changes over time were mainly due to our observations. In a first step (v1), we tested the effect of context (music vs. neutral sound) on SON/OFN discrimination, which we obtained (the cerebral response to the patient’s first name was more often observed in the music condition than in the control condition) (Castro et al., 2015). In a second step (v2), we wanted to know if externalizing the context increased the effect, which we obtained (convolution enhanced the brains’ discriminative response between SON and OFN) (Heine et al., 2021). In a third step (v3), we took for granted these two results and presented only a context externalized music to increase the sensitivity of the discriminative response detection.
  - The main aim of our study was to explore to which extent patients diagnosed with DoC were able to perform self-processing and whether the presence of this index was independent of the behavioural impairment of consciousness. Hence, we decided to average all first names (regardless of the context) to i) decrease the effects of changes across the different versions of the protocol, and ii) enhance the signal to noise ratio.
  - Interestingly, the different states of consciousness were not equally distributed among the versions of the SON paradigm (p<0.0001).
- However, the SON effects were equally distributed among the versions of the SON paradigm within each behavioural diagnostic category (p>0.05). This result emphasize that a P3 response can be identified regardless of the DoC patients’ behavioural consciousness status.
- The following figure gives a general overview of the results (number of patients experiencing the different versions of the SON paradigm (v1, v2 and v3), behavioural impairment of consciousness at the time of enrolment, presence/absence of a SON effect and clinical outcome).


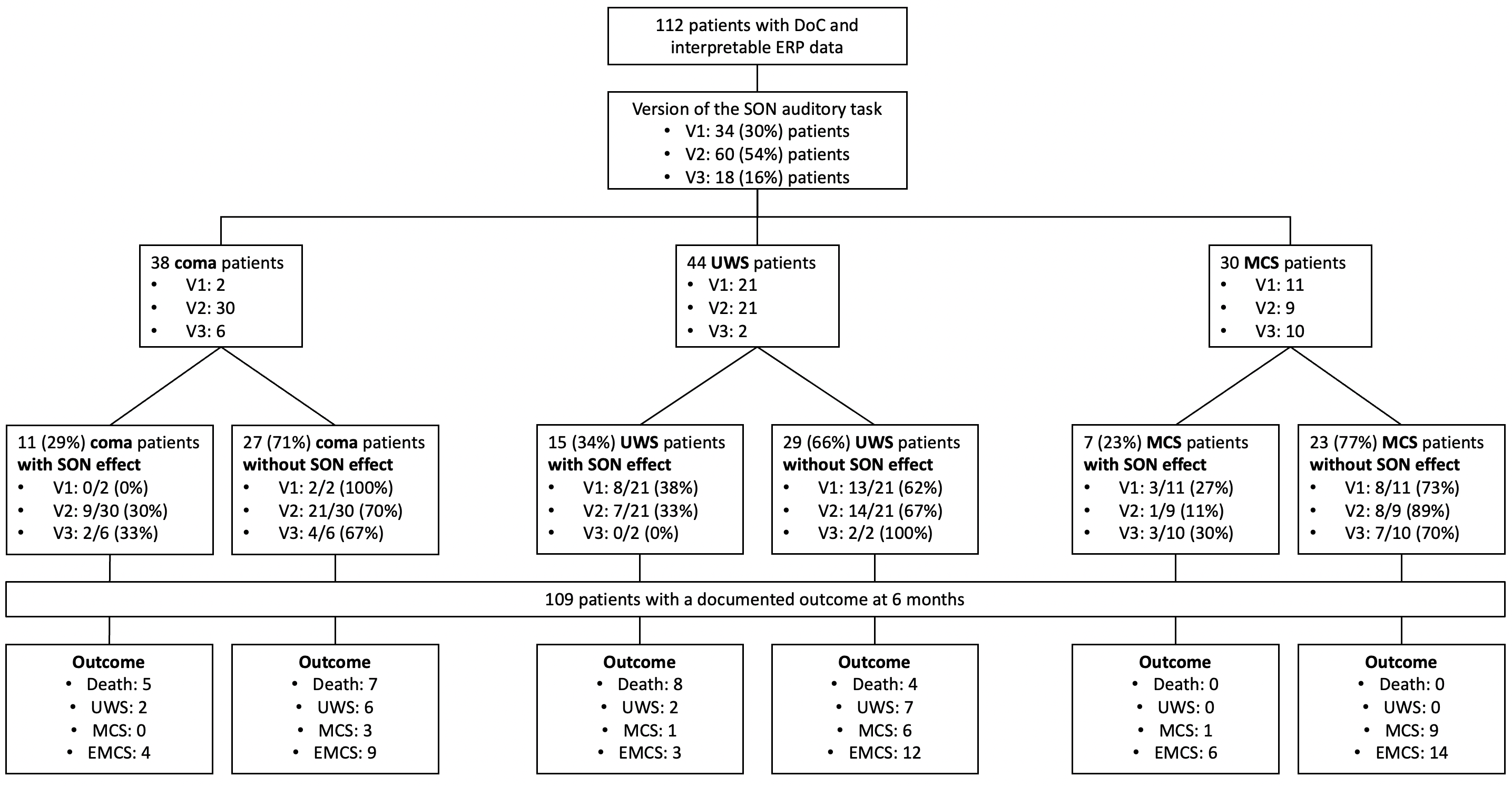


Abbreviations: DoC=disorders of consciousness; ERP=event related potentials; SON=subject’s own name; UWS=unresponsive wakefulness syndrome; MCS=minimally conscious state; EMCS=emergence from minimally conscious state

**Supplementary Figure.** Centro-parietal (Pz) grand average P3 event-related potential in a group of 22 healthy subjects in response to subject’s own name (SON) *versus* unfamiliar other first names (OFN) auditory stimuli. Using temporal clustering permutation tests, a significant effect (p<0.05) was observed from 255 ms after stimulus onset until 1000 ms (i.e., the end of the epoch).


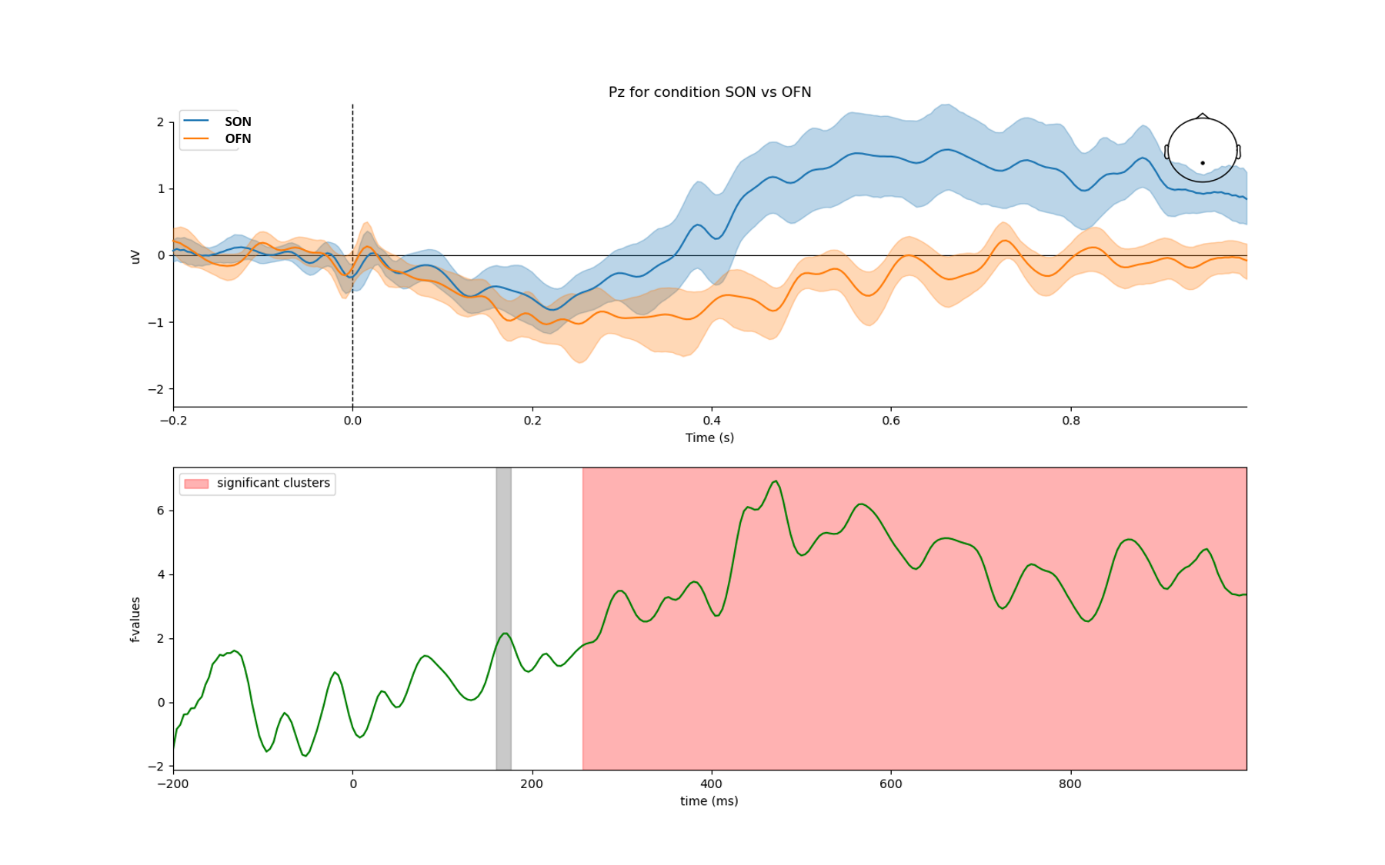


**Supplementary Table 1.** Prognostic value of a SON effect in survivors’ patients with disorders of consciousness when favourable outcome is defined by recovery of consciousness (i.e., EMCS) 6 months after brain injury.

| **Patients (n=72)** | |
| --- | --- |
| Sensitivity | 26.7% [14.6 – 41.9] |
| Specificity | 85.2% [66.3 – 95.8] |
| Positive predictive value | 75% [51.8 – 89.3] |
| Negative predictive value | 41.1% [35.5 – 46.9] |
| Likelihood ratio + | 1.8 [0.65 – 5] |
| Likelihood ratio - | 0.86 [0.68 – 1.09] |
| Area under ROC curve | 0.56 [0.44 – 0.68] |
| Patients with a SON effect | 16 (22.2%) |
| Recovery of consciousness (i.e., EMCS) at 6 months | 45 (62.5%) |
| Abbreviations: SON=subject’s own name; EMCS=emergence of minimally conscious state | |

**Supplementary Table 2.** Prognostic value of a SON effect in survivors’ patients with disorders of consciousness when favourable outcome is defined by a behavioural improvement from the time of the brain injury to 6 months after.

| **Patients (n=72)** | |
| --- | --- |
| Sensitivity | 23.6% [13.2 – 37.0] |
| Specificity | 82.4% [56.6 – 96.2] |
| Positive predictive value | 81.3% [58.3 – 93.1] |
| Negative predictive value | 25% [20.4 – 30.3] |
| Likelihood ratio + | 1.34 [0.43 – 4.15] |
| Likelihood ratio - | 0.93 [0.71 – 1.21] |
| Area under ROC curve | 0.53 [0.41 – 0.65] |
| Patients with a SON effect | 16 (22.2%) |
| Behavioural improvement at 6 months | 55 (76.4%) |
| Abbreviations: SON=subject’s own name | |

**Supplementary Table 3.** Prognostic value of the SON effect in survivors’ coma patients when favourable outcome is defined by recovery of consciousness (i.e., EMCS) 6 months after brain injury.

| **Coma patients (n=24)** | |
| --- | --- |
| Sensitivity | 30.8 % [9.1 – 61.4] |
| Specificity | 81.8 % [48.2 – 97.7] |
| Positive predictive value | 66.7 % [31 – 89.9] |
| Negative predictive value | 50 % [38.8 – 61.2] |
| Likelihood ratio + | 1.69 [0.38 – 7.55] |
| Likelihood ratio - | 0.85 [0.54 – 1.34] |
| Area under ROC curve | 0.56 [0.35 – 0.76] |
| Patients with a SON effect | 6 (25%) |
| Recovery of consciousness (i.e., EMCS) at 6 months | 13 (54.2%) |
| Abbreviations: ERP=event-related potential; SON=subject’s own name; EMCS=emergence of minimally conscious state | |

**Supplementary Table 4.** Prognostic value of a SON effect in survivors’ coma patients when favourable outcome is defined by a behavioural improvement from the time of the brain injury to 6 months after.

| **Coma patients (n=24)** | |
| --- | --- |
| Sensitivity | 25% [7.3 – 52.4] |
| Specificity | 75% [34.9 – 96.8] |
| Positive predictive value | 66.7% [31.5 – 89.7] |
| Negative predictive value | 33.3% [23.4 – 44.9] |
| Likelihood ratio + | 1 [0.23 – 4.35] |
| Likelihood ratio - | 1 [0.61 – 1.63] |
| Area under ROC curve | 0.50 [0.29 – 0.71] |
| Patients with a SON effect | 6 (25%) |
| Behavioural improvement at 6 months | 16 (66.7%) |
| Abbreviations: SON=subject’s own name | |
